# Making sense of transcribing chromatin

**DOI:** 10.1016/j.ceb.2012.02.003

**Published:** 2012-06

**Authors:** Tom Owen-Hughes, Triantafyllos Gkikopoulos

**Affiliations:** Wellcome Trust Centre for Gene Regulation and Expression, College of Life Sciences, University of Dundee, Dundee DD1 5EH, UK

## Abstract

Eukaryotic cells package their genomes into a nucleoprotein form called chromatin. The basic unit of chromatin is the nucleosome, formed by the wrapping of ∼147 bp of DNA around an octameric complex of core histones. Advances in genomic technologies have enabled the locations of nucleosomes to be mapped across genomes [1,2]. This has revealed a striking organisation with respect to transcribed genes in a diverse range of eukaryotes. This consists of a nucleosome depleted region upstream of promoters, with an array of well spaced nucleosomes extending into coding regions [2]. This observation reinforces the links between chromatin organisation and transcription. Central to this is the paradox that while chromatin is required by eukaryotes to restrict inappropriate access to DNA, this must be overcome in order for genetic information to be expressed. This conundrum is at its most flagrant when considering the need for nucleic acid polymerase's to transit 1000's of based pairs of DNA wrapped as arrays of nucleosomes.

## Highlights

► The passage of RNA polymerase is intricately coupled with chromatin alterations. ► These include the action of histone chaperones, modifying and remodelling enzymes. ► The interplay between these events is complex involving parallel pathways and feedback loops. ► Overall the process acts to ensure disruption of chromatin during transcription is transient.

## Dissociative *versus* non-dissociative models for transcribing nucleosomes

*In vitro* a range of biochemical approaches indicate that RNA polymerase II (Pol II) can pass through a nucleosome without the need for complete dissociation of histone proteins (reviewed by [3]). However the distribution of Pol II pausing sites observed *in vitro* and *in vivo* differs [4,5^••^], raising the awkward question of whether what has been observed *in vitro* accurately reflects what has taken place *in vivo*. Furthermore, changes to the experimental conditions used *in vitro* can result in increased histone dissociation. For example, closely packed polymerases are more effective in disrupting chromatin [6^•^] and it has recently been reported that transcription rates in excess of 5 bp per second result in increasing levels of histone dissociation [7^••^]. As elongation proceeds at 20 bp per second in yeast [8^••^] and up to 830 bp per second in human cells [9], dissociation of nucleosomes is a possibility. Some support for the retention of histones during elongation stems from the observation that histones retain contact with DNA at moderately transcribed genes [10–13]. However, the majority of yeast genes are transcribed sporadically, approximately seven times per hour [14^•^], making transient dissociation hard to detect. This problem is avoided at highly transcribed genes. In these cases substantial chromatin disruption is observed [15,16], but reassembly is rapid, occurring within 1 min of transcription ceasing [15]. This means that on a genome scale a correlation between histone association and Pol II occupancy could be interpreted as evidence for a transient dissociative mechanism. This is indeed what is observed [1,11–13]. What is not clear from these observations is whether Pol II is directly responsible for dissociation of histones or whether additional factors participate. Here we review the roles of some of the factors contributing to the pathway by which Pol II transits chromatin with emphasis on recent developments from studies in *Saccharomyces cerevisiae* which has proven to be an excellent model system.

## The Pol II CTD and PAF complex: recruitment platforms

Key players in the orchestration of the interplay between chromatin and transcription are the C-terminal heptapeptide repeats (CTD) of the Rpb1 subunit of Pol II and the polymerase associated PAF complex (reviewed by [17–20]). The repeated sequence (YSPTSPS) within the C-terminus of Rbp1 is subject to differential phosphorylation during different phases of the transcription cycle. It is thought to be unphosphorylated upon recruitment to promoters facilitating interactions with initiation factors such as mediator. During the early stages of elongation the CTD is phosphorylated at serine 5 (S5P) by the Cdk7 subunit of TFIIH allowing recruitment of the mRNA capping complex. CTD S5P also destabilizes interactions with initiation factors and facilitates promoter escape and recruitment of the Bur1 kinase which phosphorylates the elongation factors Spt4 and Spt5. This in turn promotes recruitment of the PAF complex comprising the Paf1, Rtf1, Cdc73, Leo1 and Ctr9 proteins. PAF and Spt4/Spt5 assist the recruitment of Rad6 and Bre1 which ubiquitinylate H2B at K123 (H2BK123Ub). H2BK123Ub is in turn required for methylation of H3K4 and H3K79 by Set1 and Dot1, respectively. Set1 itself interacts with both the PAF complex and serine 5 phosphorylated CTD and the recruitment of Dot1 is also dependent on PAF. The Bur1 and Ctk1 (P-TEFb in humans) kinases are responsible for phosphorylation of the CTD at serine 2 (S2P). This marks the polymerase for progression to a fully elongation competent form. Phosphorylation at S2P suppresses the Sen1/Nrd/Nab3 termination pathway which may contribute to the large numbers of short non-productive transcripts observed at many genes [5^••^,18]. In combination, multiply phosphorylated CTD and PAF are responsible for recruitment of the Set2 the enzyme that methylates histone H3 at K36.

## Modifications instruct modifications!

**Figure 1:**
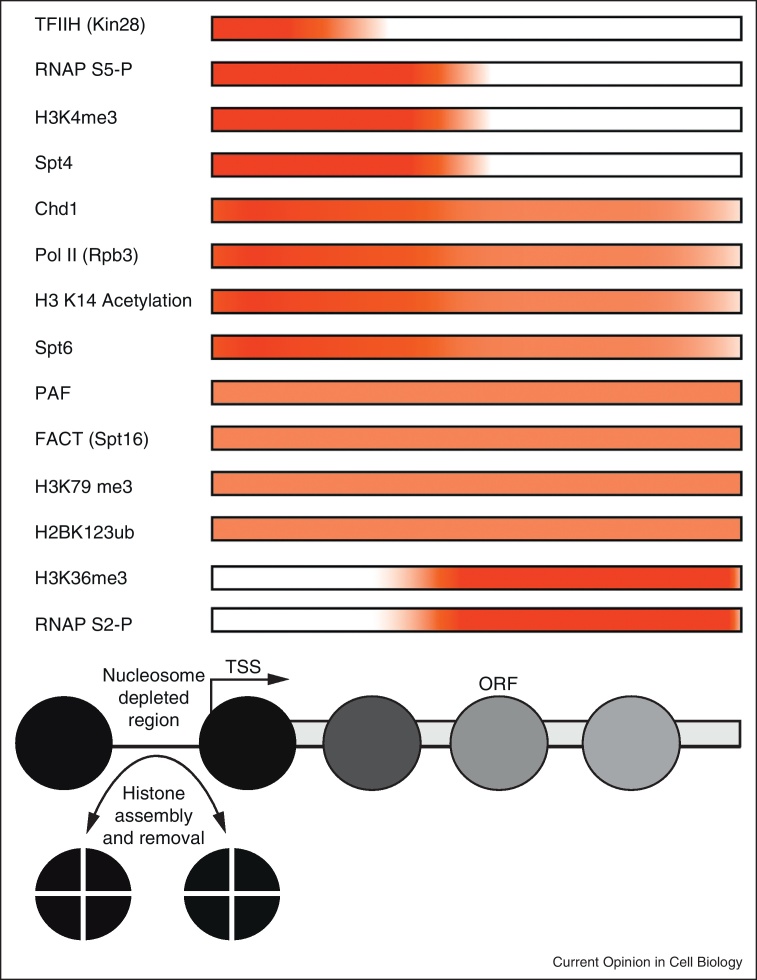
Typical distributions of histone and RNA polymerase modifications over coding regions. Genomic approaches have enabled the organisation of chromatin to be mapped at many genes. This has revealed a typical organisation for a transcribed gene involving a nucleosome depleted region upstream of the transcriptional start site followed by an array of nucleosomes. The strength of positioning decays towards the 3′ ends of genes as indicated by the lighter shading in the schematic. Enrichment for selected factors is indicated by red shading on an idealized transcribed gene and illustrates some of the interplay between chromatin and transcription. Genes transcribed at low levels do not exhibit the same pattern of enrichment, possibly reflecting the difficulty in detecting low frequency events.

One of the consequences of the pathway described above is the establishment of the characteristic distributions of histone H3 K4Me3 and H3 K36Me3 across coding regions (Figure 1). These modifications can in turn act as epitopes for the recruitment of chromatin binding proteins. For example, Eaf3 is a subunit of the histone acetyltransferase NuA4 [21] and the histone deacetylase Rpd3S [22,23]. Within Rpd3S the PHD domain of the Rco1 subunit together with the chromodomain of Eaf3 and interactions with the Pol II CTD phosphorylated at both S2 and S5 direct the complex to transcribed chromatin where it removes acetylation preventing chromatin disassembly and inappropriate initiation from within coding regions [24,25,26^•^].

An assortment of factors have been found to recognise histone H3 acetylated at lysine 4. These include the Sgf29 subunit of the SAGA complex [27^••^,28^•^]. The NuA3 HAT complex [29], human HBO1 HAT [30,31], BPTF subunit of the human NURF complex [31], the Set3c histone deacetylase complex [32] and human Chd1 [33].

The SAGA complex in addition to fulfilling a distinct function at promoters accompanies Pol II during elongation perhaps as a result of interactions between Sgf29 and H3K4Me3 and serine 5 phosphorylation of the Pol II CTD [27^••^,34,35]. This is especially prominent at highly transcribed genes such as *GAL1* where the reduced acetylation observed in the absence of SAGA is associated with increased nucleosome occupancy in the coding region and decreased mRNA production [35] especially of long transcripts [36^•^]. There is evidence to suggest that acetylated nucleosomes are targeted for removal by bromodomain containing enzymes such as SWI/SNF and RSC [34,37^•^,38]. While both SWI/SNF and RSC have functions at promoters, there is also evidence linking both complexes to elongation [37^•^,39]. Furthermore, *in vitro*, the combined effect of histone acetylation and remodelling by RSC can facilitate transcription through nucleosomes [40^•^]. However, it remains possible that there are also modes of histone dissociation independent of histone acetylation [7^••^,16].

In addition to histone acetyltransferase activity, SAGA has a deubiquitinase (DUB) activity. As a H2B Ub is required for H3 K4Me3 which in turn recruits SAGA, this enzyme has the capability to destroy the H3K4Me3 messenger that summoned its recruitment. Furthermore, the removal of H2B Ub is required for recruitment of Ctk1 and phosphorylation of Pol II at serine 2 [41^•^]. As a result the recruitment of SAGA is not only required for efficient elongation, but its association is programmed to be transient. Feedback loops of this type are exactly what is required to generate a transient wave of destabilised chromatin during transit of Poll II.

The coupling of histone acetylation mediated nucleosome dissociation with transcription potentially initiates a destabilising positive feedback loop, which could drive further nucleosome depletion and faster elongation. While this may be an advantage at genes transcribed to high levels, at genes expressed at lower levels this provides an opportunity for transcription from cryptic promoters normally occluded by chromatin. To counter this effect histone acetylation is short lived as a consequence of coupling histone deacetylase activity with transcription as described above. Where nucleosomes have been removed, chromatin assembly pathways are required to reassemble nucleosomes.

## Histone chaperones: reassembly or dissociation

Histone chaperones are prime candidates for a role in this chromatin assembly reaction [42]. Recent structural studies of the FACT chaperone complex indicate the presence of multiple domains capable of interactions with histones [43]. FACT interacts with H2A and H2B with high affinity but also interacts with H3–H4 [44], intact nucleosomes [44,45^••^] and is capable of directing the assembly of nucleosomes *in vitro* [46]. Functional studies of FACT are complicated due to the complex having distinct roles in replication and nucleosome removal at promoters. None the less, mutation of FACT results in histone depletion and increased histone exchange over coding regions and increased intragenic transcription over coding regions with no detectable change in elongation rate [47,48^•^,49]. These observations establish a role for FACT in chromatin reassembly following transcription by Pol II. This appears to contradict the original observations that FACT enhances elongation through nucleosomes *in vitro* [49]. One the one hand it could be that the original observations do not reflect the true function of FACT. Alternatively FACT may function in both the disassembly and reassembly of nucleosomes during transcription [50]. Although FACT is abundant (being present at approximately 1 copy per three nucleosomes), its action is targeted through physical interactions with the PAF complex and this requires CTD S5 phosphorylation. In addition, ubiquitin modification of H2BK123 has been observed to augment Pol II transcription through nucleosomes in the presence or absence of FACT *in vitro* [51^•^] and FACT function in chromatin reassembly *in vivo* [52^•^].

Spt6 acts similarly to FACT in the reassembly of chromatin following transcription [53^•^,54]. However, its interaction with RNA polymerase is mediated by interactions with the highly phosphorylated forms of the CTD [55–58]. Like yeast FACT, Spt6 interacts with nucleosomes only in the presence of the HMG box protein Nhp6 [45^••^,59].

The reassembly of chromatin following transcription is not restricted to coding mRNAs. Transcription of non-coding RNAs is also associated with chromatin assembly and in some cases this has been found to play regulatory roles [60–62].

## Re-phasing the template

When nucleosomes are assembled *in vitro*, in the absence of other factors, the positions adopted by nucleosomes do not fully replicate those observed *in vivo* [63,64]. An ATP dependent activity has recently been found to be capable of directing this repositioning in yeast extracts [65^••^]. Prime candidates for this include the Isw1 remodelling enzyme that has been observed to influence nucleosome spacing in mid coding regions [66] and Chd1 which interacts with the Rtf1 subunit of PAF and FACT [67]. Both Isw1 and Chd1 are found to be enriched within the coding regions of highly transcribed genes [67,68]. The genetic interactions of *CHD1* mutations with other elongation factors suggest that Chd1 acts to reduce the efficiency of elongation in a similar fashion to the Rpd3S histone deacetylase complex [69^••^]. As cryptic intragenic transcription is increased following mutation to components of either *CHD1* or *ISW1* it is possible that these proteins function with partial redundancy in chromatin assembly [69^••^]. Further support for this stems from the finding that the Chd1 and Isw1 ATPase share structurally related SANT and SLIDE accessory domains [70] and that deletion of these proteins results in an overall loss of nucleosome spacing over coding regions [68]. The establishment of regular nucleosome spacing may play an important role in stabilising the association of histones by firstly, influencing the ability of arrays of nucleosomes to form more compact structures, secondly, allowing for the association of abundant nucleosome binding proteins such as Nhp6 [71^••^] and thirdly, by simply preventing collisions between nucleosomes which can be destabilising [72]. In addition to an inhibitory effect on non-coding transcription, spaced chromatin may be less permissive to re-initiation events [73].

Some doubt remains as to how tightly coupled the nucleosome spacing reaction is to transcription. Favouring close links to transcription are the observation that nucleosome spacing decays with distance from the +1 nucleosome whose positioning is likely to be established by other factors [2], and that there are strong functional ties linking both Isw1 and Chd1 to transcription. On the other hand the spacing reaction appears to proceed in the apparent absence of transcription in nuclear extracts [65^••^] and substantial organization is retained following inactivation of RNA polymerase [74^•^]. Possible explanations for these observations include spaced chromatin being sufficiently stable to persist once established, in the absence of on-going transcription, and that there are sufficient spacing enzymes in nuclear extracts to organise chromatin in an untargeted fashion. Following inactivation of RNA polymerase a retrograde shift in the positioning of nucleosomes is observed involving many nucleosomes moving 10 bp towards the 3′ ends of coding regions [74^•^]. More recently, it has also been observed that the replacement of ancestral histones with nascent histones is slowest at the 5′ ends of long genes transcribed at low levels [75^••^]. The favoured explanation for these observations involve the net migration of nucleosomes against the direction in which RNA polymerase transcribes. As this behaviour is disrupted by deletion of the H4 tail [75^••^] which is required for the spacing activity of both Isw1 and Chd1, it is tempting to speculate that spacing in the wake of a transcribing polymerase is associated with a net movement of nucleosomes in a 5′ direction.

## The problem with elongation

The very fact that Pol II moves across genes during elongation complicates studies of its localisation in comparison to activities which are recruited to fixed loci such as promoters. Furthermore, many genes are actively transcribed for relatively short periods of time, making it even harder to study factors involved in elongation using chromatin immunoprecipitation assays. Genetic studies of elongation factors are complicated as a result of them often having distinct roles in other processes such as chromatin reconfiguration at promoters (*e.g.* FACT, SAGA, SWI/SNF, RSC, NuA4, Chd1 and Isw1). Where a strong phenotype is conferred by one mode of action, for example promoter remodelling, it may confuse interpretation relating to transcriptional elongation. Another reoccurring issue is the presence of parallel pathways that confer partial redundancy which greatly complicates the interpretation of genetic interactions. For example, due to overlapping functions, multiple HDACs and chromatin remodelling enzymes must be removed to observe defects [26^•^,68].

## The logic of the transcription cycle

**Figure 2:**
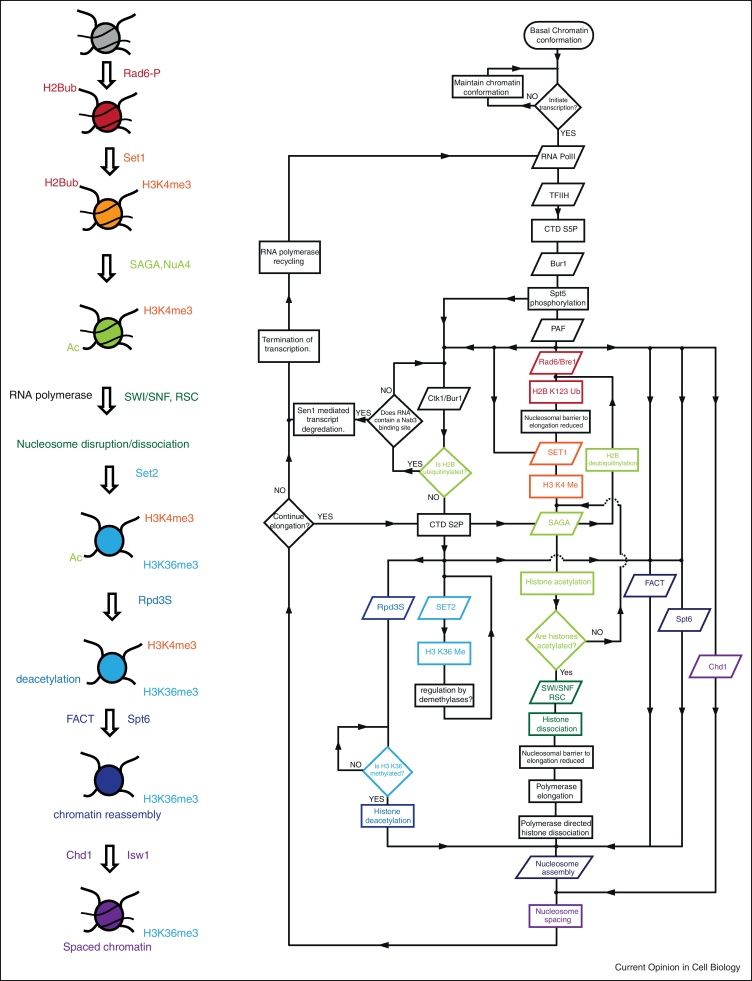
Systematic representation of selected events occurring during elongation through chromatin. The left panel summarises the series of alterations to chromatin occurring during the course of transcriptional elongation that are discussed in the text. In the right panel an attempt has been made to integrate these events with other events occurring during transcriptional elongation in the form of a flow diagram. Diamond shaped symbols represent decisions, rectangles represent processing steps, parallelograms input or output (normally recruitment of a complex), connected by lines with information flowing in the direction indicated by arrows. The colouring used in both panels is the same. The diagram is far from comprehensive as some aspects have been simplified or omitted to retain clarity. For example the diagram incorporates the SAGA, and Rpd3s HAT and HDAC complexes, but it is known that other HAT's (*e.g.* NuA4 [34]) and HDAC's (*e.g.* Set3C [26]) also function in elongation, and others may be as yet unidentified. Despite these limitations it is clear that the process involves parallel pathways and feedback loops. Similar features have been identified using systematic approaches [79,90]. For example H2B ubiquitination is required for H3K4 methylation, which in turn recruits SAGA which can remove the H2BK123Ub mark. Furthermore removal of H2BK123Ub is required for conversion of Pol II to the fully elongation competent form phosphorylated at serine 2. This form of RNA polymerase can act to recruit the Rpd3S histone deacetylase, which is capable of reversing histone acetylation deposited moments earlier by the SAGA (or NuA4) complexes. This logic is ideally suited to ensuring that destabilisation of chromatin during transit by Pol II is both tightly coupled to transcription and transient.

Over the last decade important insights into many of the factors involved in transcribing through chromatin provide the opportunity to take a step back and consider the overall organisation of the pathway (Figure 2). The pathway involves branching and feedback connections that act to ensure process such as H2BK123Ub and histone acetylation not only occur during transcription, but are also transient. The overall logic of the process appears to be largely directed at ensuring the processes of chromatin disassembly and reassembly are tightly coupled with transcription in a fashion that is comparable with the cell cycle where multiple check points ensure regulated progression with the single outcome of duplication. Many of the chromatin related factors involved in transcriptional elongation have roles in chromatin reassembly following transcription. Their action may be directed at breaking the potentially dangerous positive feedback loop that could result if a pioneering polymerase disrupts chromatin so as to facilitate subsequent transcription events from both the coding and non-coding strands. This would be expected to result in correlated bursts of transcription, an effect that appears not to occur at typical yeast genes transcribed at moderate levels [8^••^]. However, this situation may differ at more highly regulated genes where short burst of transcription have been observed [76], and there may be a greater requirement for memory effects in organisms with more complex developmental programmes [77].

Highly transcribed genes are observed to be enriched for distinct patterns of histone modifications (Figure 1). The different distributions of these modifications can largely be attributed to differences in the frequency with which RNA polymerase transcribes a gene. For example, at genes transcribed at low levels Pol II directed histone acetylation may be short lived as a result of coupled action of histone deacetylases. Indeed, chromatin reassembly following transcription has been estimated to occur within 1 min [15]. In contrast, the deacetylated and methylated state may be relatively stable. As yeast genes are transcribed at an average rate of seven transcripts per hour [14^•^], most genes would be expected to be reassembled as chromatin 90% or more or the time. However, at very highly expressed genes, the high frequency of polymerase passage would be expected to dramatically increase the proportion of time chromatin is disrupted. Overall, the application of this process to large numbers of genes provides a means of directing many of the observed patterns of histone modification across coding regions (Figure 1). Removal of methylation marks by histone demethylases is an area that requires further investigation. Evidence to date suggests that demethylases are likely to be involved, but are as yet difficult to place in the overall pathway [78^••^]. Furthermore dilution of methylation marks during replication may play a role in the removal of H3K4 methylation [78^••^]. This is in effect the reverse of the idea that histone marks are stably inherited from one generation to the next. As a result, it seems likely that a significant proportion of histone modifications that characterise the coding regions of yeast genes do not comprise an epigenetic signal, but are instead instructed by the frequency of transcription.

The above description is no doubt a simplification. There is considerable variation in the ways that different genes respond to the loss of different component's of the transcriptional machinery [79]. There is also evidence that the elongation machinery is deployed in different ways at different yeast genes [26^•^,53^•^], and this is clearly deployed as a major point of regulation in higher eukaryotes [80–84]. However, many of the histone modifications associated with elongation appear to function in a similar way at large numbers of genes. As a result, they are not acting to specify a broad range of distinct downstream functions as proposed in the histone code hypothesis [85].

In summary, the process of transcription through chromatin is becoming clearer as a result of a huge effort to characterise each of the steps involved. While there are undoubtedly many aspects that still remain to be discovered, overall the process provides a means of ensuring that the dynamic events occurring at the site of transcription are restored. In this way a substantial proportion of the chromatin landscape can be considered as being directed towards discretely covering the tracks left by the passage of RNA polymerase.

## References and recommended reading

Papers of particular interest, published within the period of review, have been highlighted as:

• of special interest
•• of outstanding interest

## Figures and Tables

Data adapted largely from [68,86,87^•^,88,89^•^].
